# Purine-rich element binding protein alpha: a DNA/RNA binding protein with multiple roles in cancers

**DOI:** 10.1186/s10020-025-01087-8

**Published:** 2025-01-22

**Authors:** Shiyi Yu, Chengyang Jiang, Yawen Yang, Fei Cheng, Fangchen Liu, Chang Liu, Xue Gong

**Affiliations:** 1 Institute of Translational Medicine, Medical College, Yangzhou University, Yangzhou, Jiangsu China; 2 Jiangsu Key Laboratory of Experimental & Translational Non-Coding RNA Research, Yangzhou University, Yangzhou, Jiangsu China; 3 Nanjing Women and Children’s Healthcare Hospital, Maternal and Child Health Institute, Women’s Hospital of Nanjing Medical University, 123 Tianfei Alley, Mochou Road, Nanjing, China

**Keywords:** DNA/RNA binding protein, PURα, Cancer, Biomarker

## Abstract

Proteins that bind to DNA/RNA are typically evolutionarily conserved with multiple regulatory functions in transcription initiation, mRNA translation, stability of RNAs, and RNA splicing. Therefore, dysregulation of DNA/RNA binding proteins such as purine-rich element binding protein alpha (PURα) disrupts signaling transduction and often leads to human diseases including cancer. PURα was initially recognized as a tumor suppressor in acute myeloid leukemia (AML) and prostate cancer (PC). Most recently, several studies have revealed that PURα is dysregulated in multiple cancers, such as breast cancer (BC) and esophageal squamous cell carcinoma (ESCC). The oncogenic or tumor-suppressive functions of PURα are realized via regulating RNA/protein interaction, mRNA translation, formation of stress granules (SGs), and transcriptional regulation of several oncogenes and tumor suppressors. Although DNA/RNA binding proteins are hardly targeted, novel strategies have been applied to identify compounds targeting PURα and have demonstrated promising anti-tumor efficacy in the preclinical study. The present review summarizes the most recently discovered critical roles of PURα in various cancer types, providing an overview of the biomarker and therapeutic target potential of PURα for patients with cancer.

## Introduction

The human Purine-rich element-binding protein alpha (PURα) was first identified in 1992 by Bergemann, by deciphering DNA binding proteins of the purine-rich region in the c-Myc promoter (Bergemann and Johnson 1992). *PURA* is an evolutionarily conserved gene, with a similarity of > 99% in the encoded protein between humans and mice (Ma et al. 1994). Since then, studies have focused on revealing PURα as a transcription factor in neurons, such as PURα stimulates the transcription of MBP in oligodendrocytic cells (Zambrano et al. 1997; Haas et al. 1995; Chen and Khalili 1995). Later, in 1998, Chepenik identified the interaction between PURα and RNA, which is critical for the self-association and DNA-binding activity of PURα (Chepenik et al. 1998). Further studies focus on unveiling how PURα regulates neural development and viral transcription, demonstrating that the direct interaction between RNA and PURα favors the transcription from DNA, promotes RNA-templated transcription, and enhances mRNA translation (Tretiakova et al. 1998; Gallia et al. 1999, 2001; Kobayashi et al. 2000).

Cancer is initiated by oncogenic cells which are featured with an array of attributes including genetic mutations and epigenetic dysregulation, eventually leading to the expansion and spread of tumor cells (Hanahan and Weinberg 2011). In 2001, deletion of the *PURA* loci was observed in a majority of patients with myeloid disorders, suggesting its involvement in the progression of myelodysplastic syndromes (MDS) and acute myelogenous leukemia (AML) (Lezon-Geyda et al. 2001). Later, PURα has been identified as a potential tumor suppressor in several cancer types, including glioblastoma (GBM), prostate cancer (PC), and breast cancer (BC) (Adema et al. 2022; Darbinian et al. 2001; Inoue et al. 2008; Yu et al. 2023; Wang et al. 2008). However, in 2021, Gao discovered high expression of PURα in esophageal squamous cell carcinoma (ESCC) tumors and identified a critical role of PURα in promoting the metastasis of ESCC cells via transcriptional activation of Snail2 (Gao et al. 2021). Later, several reports also demonstrated the oncogenic role of PURα in ESCC (Tian et al. 2022; Xu et al. 2023). PURα is a well-characterized DNA/RNA binding protein critical for nucleic acid transport and direct or indirect regulation of gene transcription (Johnson et al. 2013). Several recent studies have uncovered novel molecular mechanisms of PURα in cancer cells which involved in cancer progression. For example, as a novel biological function, PURα has been discovered to organize stress granules in the ESCC cells (Tian et al. 2022). Furthermore, new interacting partners have been revealed in cancer cells, including non-coding RNAs and proteins critical for cancer progression (Yu et al. 2023; Xu et al. 2023). The present review delivers a comprehensive summary of several facets of PURα, including its aberrant expression patterns in cancer, the regulations concerning its expression and activity, the core molecules responsible for the role of PURα in cancer cells, and the targeted approaches for PURα.

## Structure of PURα protein

**Fig. 1:**
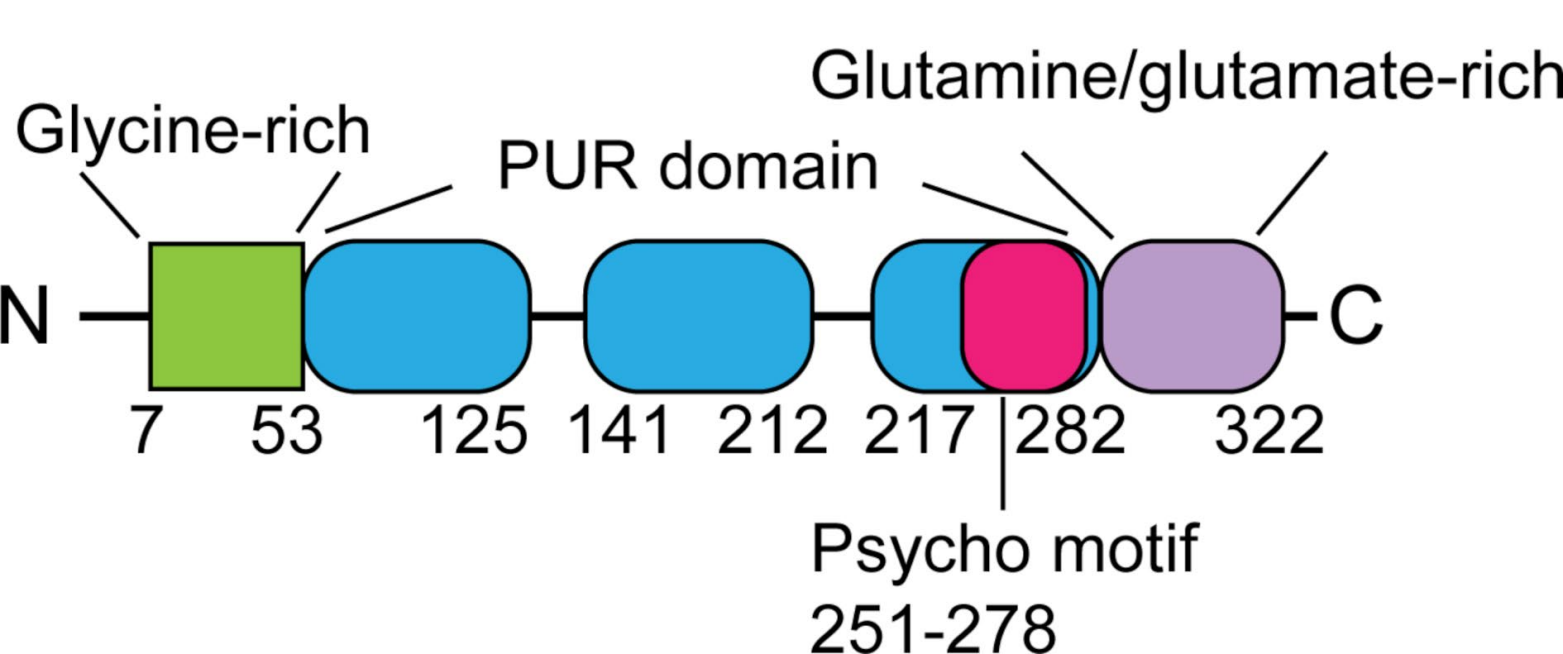
Schematic representation of PURα protein structure The PURα protein contains the glycine-rich, PUR, and glutamine-glutamate-rich domains. The N-terminal glycine-rich domain is involved in protein-protein interaction. The PUR domain is critical for DNA/RNA binding which includes a psycho motif for interaction with pRb protein. The glycine-rich domain is potentially pivotal for the transcriptional activation function of the PURα protein

Analysis of the 322 amino acids of PURα protein revealed several structural regions including the glycine-rich domain, the PUR domains, the psycho motif, and the glutamine-glutamate-rich domain (Gallia et al. 2000) (Fig. 1).

The strongly conserved PUR domain is the most critical structure for the function of PURα protein. The PUR domain is around 80 amino acids in length, conferring DNA/RNA binding ability for the protein (Bergemann and Johnson 1992). According to the crystal structure of *D. melanogaster* PURα, the PUR domains were constituted by either the interaction between repeat I and repeat II or 2 repeats III to form an inter-molecular homodimer (Weber et al. 2016).

Despite the central-localized PUR domain, there are also several important structural features in the PURα protein. The glycine-rich domain is located in residue 7–53. There is a stretch of glycine in the region disrupted by only 1 serine residue. The glycine-rich domain is supposed to be involved in protein-protein interaction (Bossemeyer 1994). The psycho motif refers to the consensus sequences found in proteins containing PSY and C. The psycho motif is found in the 251–278 residue of the PURα protein, which mediated its interaction with pRb protein (Johnson et al. 1995). Half of the residues in the PURα protein region from 282 to 322 are glutamine or glutamate, called the glutamine-glutamate-rich domain. This region is potentially pivotal for the transcriptional activation function of the PURα protein (Tjian and Maniatis 1994).

## Aberrant expression of PURα protein in liquid and solid cancers and its prognostic value as a biomarker

Studies have revealed aberrant expression of PURα in specific cancer types e.g., low PURα expression or gene deletion was observed in AML, Myeloid neoplasia (MN), hormone-refractory prostate cancer (HRPC), and BC (Lezon-Geyda et al. 2001; Inoue et al. 2008; Yu et al. 2023), and high PURα expression was observed in ESCC (Gao et al. 2021) (Table 1). In addition, PURα was identified as an indicator of prognosis and was associated with drug sensitivity for patients with BC and ESCC (Yu et al. 2023; Gao et al. 2021).

**Table 1:** PURA is dysregulated in cancers MDS: myelodysplastic syndromes; AML: Acute myeloid leukemia; MN: myeloid neoplasia; PC: prostate cancer; BC: breast cancer; ESCC: esophageal squamous cell carcinoma; IHC: immunohistochemistry; WB: Western blotting

Cancer type	Type of evidence	PURA status	Mechanism (Dysregulation)	PURA function	Mechanism (Function)	Reference
MDS and AML	FISH of 60 patient specimens	Downregulated	Gene deletion	Unknown	Unknown	12
MN	RNA-Seq of 995 patient samples	Downregulated	Gene deletion	Unknown	Unknown	13
PC	IHC of 36 patient specimens	Downregulated	Unknown	Tumor suppressor, negatively control androgen-independent cell growth	Transcriptional repression of AR expression	17
BC	IHC of 70 tumors and 4 normal tissues, WB of 14 pairs of tumors and normal tissues	Downregulated	Unknown	Tumor suppressor, inhibit cell cycle progression	Inactivation of E2F1 via direct binding	16
ESCC	IHC of 255 patient specimens	Upregulated	Unknown	Oncogene, promote cell proliferation and metastasis	Transcriptional activation of Snail2 expression	18
ESCC	IHC of 282 pairs of tumors and nontumorous epithelia	Upregulated	Unknown	Oncogene, promote cell proliferation and metastasis	Suppression of IGFBP3 expression via binding to 3’UTR of mRNA	19

### PURα loss in cancers

Chromosome deletions are one of the most well-characterized hallmarks of cancers (Hanahan and Weinberg 2011). For MDS and AML, deletions of chromosome 5 are frequently observed in clinical samples. Using Fluorescence In-Situ Hybridization (FISH), Kimberly revealed that the *PURA* locus (q31.1 band on chromosome 5) was often hemizygous deleted in sixty patients diagnosed with MDS or AML. Consistently, PURα was lowly expressed in the bone marrow cells of MDS or AML patients (Lezon-Geyda et al. 2001). Most recently, by analyzing clinical data of patients, another study also revealed that the deletion of the *PURA* gene in MDS was one of the anti-drivers for the pathogenesis of MDS, and was also associated with the clinical phenotype and poor prognosis of MDS patients (Adema et al. 2022).

The development of androgen independence is a major threat for patients with PC, due to its contributions to hormone therapy resistance (Dai et al. 2023). By exploring the Oncomine database, PURα mRNA levels were significantly lower in HRPC samples than in Hormone-naive Prostate Cancer (HNPC) samples in a large cohort (Wang et al. 2008). Additionally, immunohistochemistry (IHC) staining at protein levels showed that the nucleus-localized PURα protein was significantly lower in HRPC samples than in HNPC samples (Wang et al. 2008).

BC is the most prevalent cancer type for women worldwide. Via analyzing databases, our previous study discovered that PURα exhibits lower expression in BC at both transcriptional and translational levels (Yu et al. 2023). IHC staining showed that PURα protein levels were negative for most tumor samples of various subtypes. The immunoblotting further confirmed the downregulation of PURα protein levels in 14 pairs (92.9% tested cases) of BC tumors compared with the matched normal tissues (Yu et al. 2023). Additionally, low expression of PURα is associated with poor overall survival (OS) and relapse-free survival in BC patients (Yu et al. 2023).

### Upregulation of PURα in ESCC

Due to distant metastasis, the prognosis of ESCC patients is poor with a 5-year OS rate of 15% (Jeong et al. 2020). In 2021, the study by Gao demonstrated for the first time that the high expression of PURα was positively correlated with lymph node metastasis and advanced AJCC stage of ESCC via IHC staining of 255 ESCC samples, which suggested that PURα was critical for the progression of ESCC (Gao et al. 2021). After that, Tian identified a profound elevation of cytoplasmatic PURα protein in ESCC compared with matched normal tissues by IHC staining of 282 cases (Tian et al. 2022). Furthermore, a Kaplan-Meier survival analysis shows poor OS for those exhibiting increased cytoplasmic expression of PURα compared with those with low cytoplasmic expression of PURα in a large cohort (526 ESCC cases), indicating PURα as a potential prognostic biomarker for ESCC (Tian et al. 2022).

## Regulation of PURα expression and activity

### Regulation of PURα expression

**Fig. 2:**
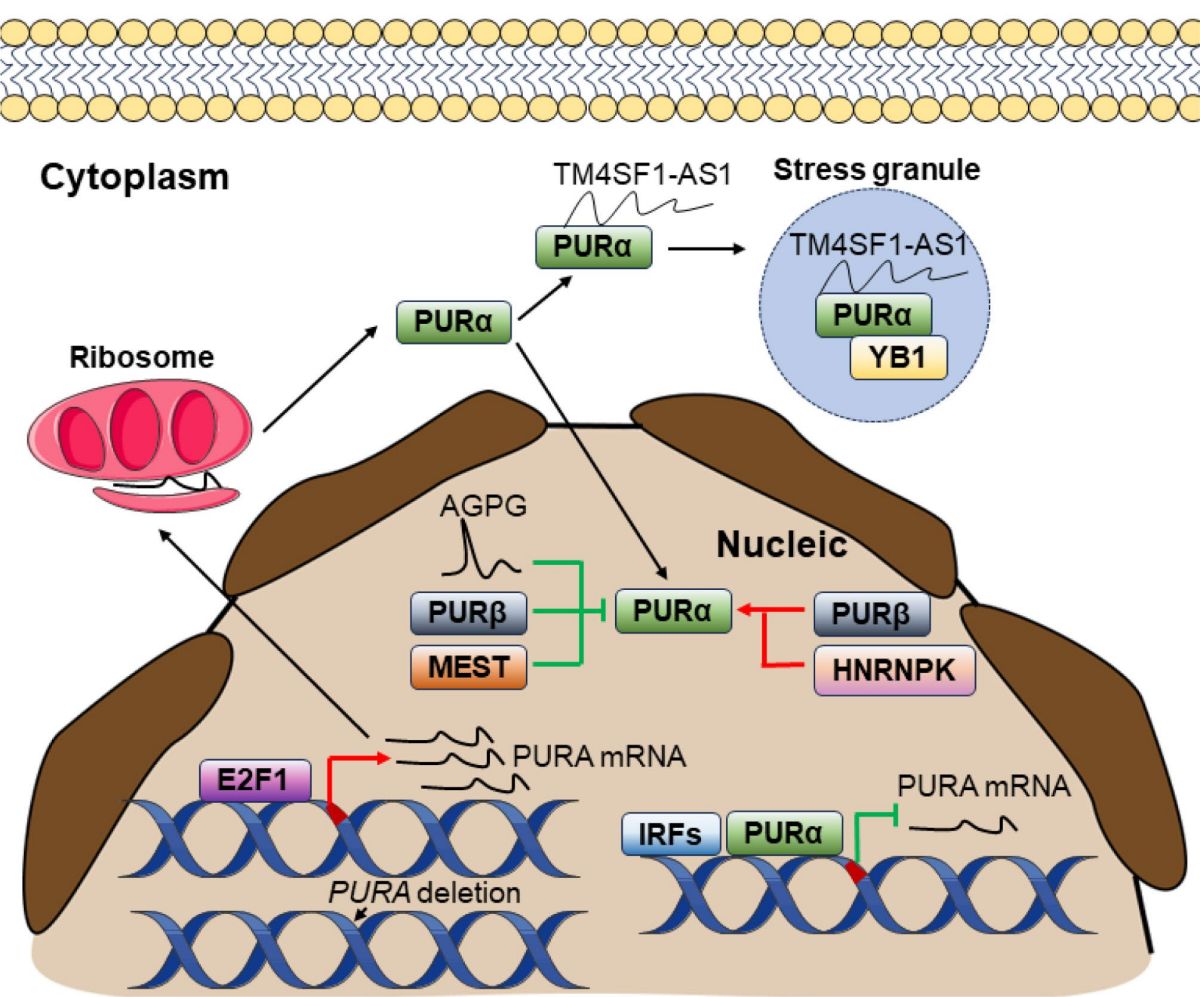
Schematic representation of molecular mechanisms driving dysregulation of PURα expression and activity In the nucleic fraction, the PURα mRNA level is regulated by gene deletion and transcription factors (PURα, IRFs, and E2F1). After translation, the activity of nucleic localized PURα protein is regulated by the direct binding of AGPG, PURβ, and HNRNPK in cancer cells. In the cytoplasmic fraction, PURα protein interacts with TM4SF1-AS1 and YB1, organizing stress granules, and showing anti-apoptosis activity in cancer cells

Our understanding of the upstream pathways that regulate PURα protein expression is limited. It has been observed in several cancer types about the dysregulation of *PURA* mRNA levels, is attributed to chromosome deletion in AML and MDS (Lezon-Geyda et al. 2001; Adema et al. 2022). Some of the transcription factors which currently influence PURα protein expression are shown in Fig. 2. Interestingly, the fine control of *PURA* mRNA levels involves self-regulation mechanisms (Darbinian et al. 2006; Muralidharan et al. 2001). Human *PURA* has 3 transcription start sites (TSSs) which are regulated by different transcription factors. Among them, human *PURA* TSS I is very homologous to mice (Wortman et al. 2010). In human and mouse cells, approximately 6,000 bp upstream of the TSS I of *PURA* exhibits promoter activity. The promoter contains several GC/GA-rich motifs that facilitate PURα protein binding. Ectopic expression of PURα decreased the activity of PURα promoter, suggesting the autoregulation of PURα expression at the transcription level.

Analysis of the promoter region also revealed that the transcription of *PURA* is regulated by one of the binding partners of PURα, i.e., E2F1. In GBM cells, E2F1 can bind to a specific E2F1 consensus binding motif on the *PURA* promoter, thus activating the *PURA* transcription and increasing PURα expression (Darbinian et al. 2006). The association of pRb with E2F1 can inhibit E2F1 activity, thereby dampening the activation of the *PURA* promoter (Darbinian et al. 2006).

Different from TSS I, TSS II and TSS III are located near the translational start codon. Like the TSS I transcript, transcription of TSS III is elevated by E2F1. In contrast, several elements critical for innate immunity are near TSS II, and IRFs negatively regulate its transcription.

### Regulation of PURα activity

Accumulating studies indicate that the activity of PURα protein is tightly controlled by several of its binding partners. The *PURB* gene encodes PURβ, which shares 70% sequence conservation with PURα (Johnson et al. 2013). PURα is ubiquitously expressed in human tissues, however, PURβ expression is only detected in several certain human tissues (Johnson et al. 2013). Studies demonstrate that complexes involving PURα and PURβ regulate the transcription initiation of several critical genes in muscle cells, such as alpha-actin (ACTA1) and myosin heavy chain gene 7 (MYH7) (Kelm et al. 1997; Ji et al. 2007). In primary cardiac myocytes, PURβ exhibits a dominant negative effect on the activity of PURα by transcriptionally regulating α-MHC gene expression (Gupta et al. 2003). However, PURβ also strongly enhances the ability of PURα/YB-1 to compete with p-SMAD3 on the ACTA2 promoter (Hariharan et al. 2014). Altogether, these studies manifest that the activity of PURα is enhanced or decreased by PURβ in different backgrounds.

Elevated expression of androgen receptor (AR) is critical for the development of androgen-independent PC (Wang et al. 2008). Investigation of the AR suppressor element nuclear binding complex identifies PURα and HNRNPK as core factors in the complex. In PC cells, PURα and HNRNPK show a synergistic effect on the repression of AR transcription. Silencing of HNRNPK enhances the effect of PURα silencing on AR reporter activity in androgen-independent PC cells.

In ESCC cells, immunoprecipitation (IP) and mass spectrometry (MS) analysis identified mixed epithelial and stromal tumor (MEST) as a novel binding partner of PURα (Xu et al. 2023). The binding of MEST to PURα blocked the physical interaction between PURα and the promoters of *SRCIN1* and *RASAL1*, leading to the activation of ERK signaling and cancer metastasis (Xu et al. 2023). Via molecular docking, a compound (G699-0288) targeting the interaction between MEST and PURα has been identified, and G699-0288 inhibits cancer cell metastasis in vivo.

Most recently, our study has shown that lncRNA AGPG disrupted the interaction between PURα and E2F1 in the nucleus via physical interaction with PURα protein in the context of BC (Yu et al. 2023). The physical interaction between AGPG and PURα is facilitated by the loop domains of AGPG and the DNA/RNA binding domain of the PURα protein. The binding of AGPG to PURα resulted in the dissociation of PURα from the PURα/E2F1 complex, which dampened the effect of PURα on the activation of the E2F1 signaling pathway, and intensified the endocrine resistance of BC cells.

Stress granules (SGs) are membrane-less organelles composed of RNA and proteins that are formed by cells under stress conditions, and they play a crucial role in managing cellular stress responses and suppressing apoptosis in cancer cells (Kitajima et al. 2023). Kitajima has uncovered that the interaction between TM4SF1-AS1 and PURα promoted the formation of SGs, inactivated stress-responsive mitogen-activated protein kinases (MAPK) signaling, and inhibited cellular apoptosis in gastric cancer (GC) cells (Kitajima et al. 2023).

## Mechanisms of PURα in tumorigenesis

**Fig. 3:**
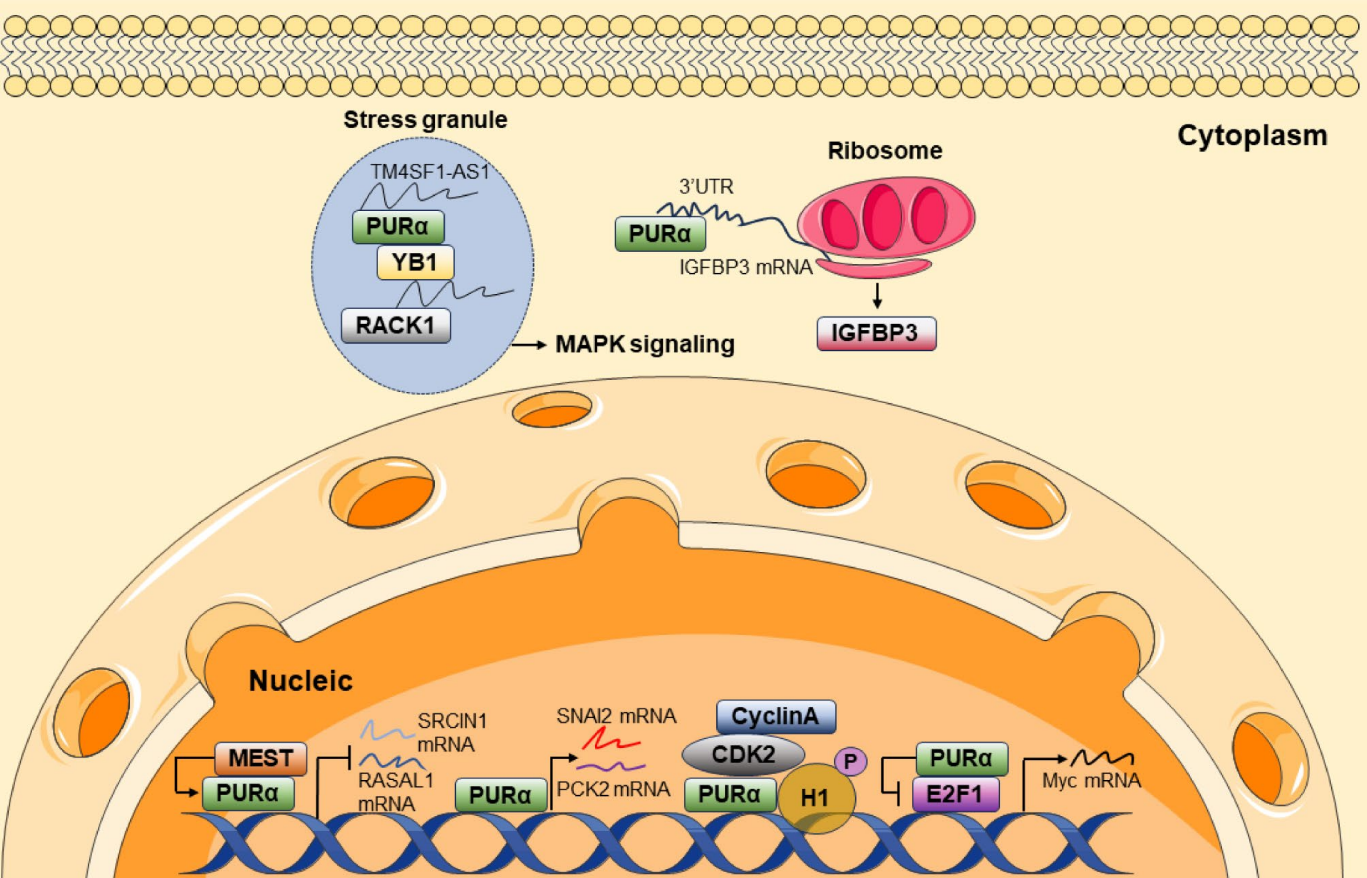
PURα protein organization and signaling networks in cancers Cancer-relevant PURα interacting proteins and the direct downstream target RNA and DNA are shown. In the nucleic fraction, PURα transcriptionally activates or represses target gene expression via directly binding to DNA or forming complexes with transcription factors or CDK2/CyclinA. In the cytoplasm fraction, PURα organizes the stress granules to activate the MAPK pathway. PURα also binds to 3’UTR of IGFBP3 mRNA to promote translation

For the past 5 years, studies emphasized several oncogenes as direct targets of PURα and have elucidated that the regulation is critical for cancer development. Despite the well-known functions of PURα as a transcription co-factor and transcription factor, most recent studies also demonstrated the novel roles of PURα in the development of cancers, such as regulating translation initiation of mRNA and orchestrating stress granules (Fig. 3).

### PURα functions as a transcription co-factor

Uncontrolled accelerated cell cycle progression is a hallmark of cancer. PURα can bind to a variety of Cyclin/cyclin-dependent kinase (CDK) complexes, including Cyclin A/CDK2, Cyclin B1/CDK1, p35/CDK5, Cyclin T1/CDK9, and CDK5-containing complexes, contributing to cell cycle progression (Liu et al. 2005). In cervical cancer (CC), PURα directly bound to CDK2 and activated the transcription of oncogene c-Myc. Mechanistically, CDK2 itself could not bind to DNA. The formation of the PURα/CDK2/Cyclin A complex conferred the DNA binding ability and stimulated the phosphorylation of histone H1 to promote c-Myc transcription.

E2F1 is a transcription factor that plays a crucial role in regulating the cell cycle (Darbinian et al. 2006). E2F1 can form a ternary complex with PURα on specific DNA fragments in the initiation region of DNA replication (Darbinian et al. 2006). Association with PURα strongly suppresses the ability of E2F1 to initiate the transcription of cell-cycle-related genes and affects the occurrence and development of cancer (Darbinian et al. 2001). In BC cells, PURα negatively regulates the expression of cell-cycle-related genes. Knockdown of lncRNA AGPG disrupts the formation of the PURα/E2F1 complex, releasing E2F1 from the PURα/E2F1 complex, leading to the activation of the E2F1 signaling pathway, which promotes BC cell cycle progression and endocrine resistance (Yu et al. 2023). Caspase 8 (CASP8) is a critical initiator of apoptosis and is dysregulated in various cancers, including ESCC (Weber et al. 2016). In ESCC cells, PURα interacts with E2F1 at the promoter regions of the CASP8 gene and initiates CASP8 transcription (Lin et al. 2014). These studies collectively demonstrated that physical interaction between E2F1 and PURα inhibited the transcription activation activity of E2F1 and was critical for the tumor suppressive function of PURα.

### PURα functions as a transcription factor

Several studies discovered that PURα can act as a transcription activator or transcription repressor to regulate PCK2, Snail2, SRCIN1, and RASAL1 mRNA expressions in ESCC cells directly, thus promoting metastasis (Gao et al. 2021; Xu et al. 2023; Sun et al. 2020).

Through bioinformatics analysis of RNA-seq and ChIP-seq data, one study found that PURα affects metabolic pathways, including oxidative phosphorylation and fatty acid metabolism in ESCC cells (Sun et al. 2020). Specifically, PURα binds to the GGGAGGCGGA motif in the promoter of the PCK2 (Sun et al. 2020). The protein and mRNA levels of PCK2 were enhanced by PURα overexpression and suppressed by PURα knockdown in ESCC cells (Sun et al. 2020).

Snail2 is a transcription factor regulating the occurrence of epithelial-mesenchymal transition (Gao et al. 2021). By constructing a luciferase reporter containing the Snail2 promoter sequence, PURα was found to bind to a specific region of the Snail2 promoter and enhance its transcriptional activity (Gao et al. 2021). PURα promotes the epithelial-mesenchymal transition process in ESCC cells by enhancing the transcription of Snail2, leading to the loss of intercellular junctions and increased cell invasion ability (Gao et al. 2021).

Extracellular regulated protein kinases (ERK) pathway is a well-characterized oncogenic signaling in cancer cells. In ESCC cells, a recent study has discovered that PURα repressed the expression of SRCIN1 and RASAL1, 2 negative regulators of the ERK pathway. PURα binds to the PURA-responsive elements in the promoter regions of SRCIN1 and RASAL1 to suppress their transcription. MEST directly interacts with PURα in ESCC cells, thus favoring the function of PURα in repressing SRCIN1 and RASAL1 transcription (Xu et al. 2023). The MEST-PURα interaction is pivotal for the activation of SRCIN1/RASAL1-ERK-Snail signaling and cancer metastasis (Xu et al. 2023).

### PURα controls mRNA translation

As an RNA-binding protein, PURα also plays a crucial role in cancer progression by interacting with mRNA, mainly through influencing its translation (Tian et al. 2022). In ESCC cells, PURα predominantly binds to the 3’ untranslated region (3’UTR) of mRNA, as revealed by Cross-Linking and Immunoprecipitation sequencing (CLIP-seq) analysis. Moreover, PURα directly interacts with translation initiation factors, such as PABPC1, eIF3B, and eIF3F, and ribosome-associated proteins, suggesting the potential of PURα in regulating mRNA translation. For example, PURα binds to the 3’UTR of Insulin-Like Growth Factor Binding Protein 3 (IGFBP3) mRNA, thereby inhibiting the initiation of IGFBP3 mRNA translation. IGFBP3 silencing partially rescued the anti-metastasis and anti-proliferation function of PURα knockout, suggesting that the regulation of mRNA translation is crucial for the oncogenic role of PURα in ESCC.

### PURα organizes SGs

The formation of SGs supports the cancer cells to survive under stress (Biancon et al. 2022). A recent study found that TM4SF1-AS1, PURα, and YB-1 proteins are located in the cytoplasm of stress granules and form a complex to sequester RACK1 protein in the SGs. Consequently, the stress-responsive MAPK signaling pathway was activated to inhibit cell apoptosis in GC cells (Kitajima et al. 2023).

## Targeting PURα for patients with cancer

The mutation and aberrant expression of PURα contribute to cancer progression via multiple mechanisms including directly or indirectly regulating gene expression, initiating translation, and SGs formation, making it a promising therapeutic target for cancers. Unfortunately, as a transcription regulator and RNA binding protein, PURα is traditionally “undruggable” (Xie et al. 2023). In recent years, however, disrupting the binding of PURα to its interacting partners has been discovered as an alternative approach to target PURα (Xu et al. 2023). Moreover, applying advanced biological techniques also uncovered a novel PURα agonist from natural compounds (Chen et al. 2023).

Xu has found that the physical interaction between MEST and PURα promoted ESCC metastasis (Xu et al. 2023). They used the molecular docking method to discover compounds that could target amino acids 138–142 of MEST protein in a large compound library. The candidates were further screened with an ELISA assay and co-IP assay to identify small molecules that could disrupt the interaction between MEST and PURα. G699-0288 was found to block the binding of MEST to PURα, thus inactivating the ERK pathway in ESCC cells (Xu et al. 2023). More importantly, tail vein injection of G699-0288 strongly inhibited tumor metastasis in a PDX model, whose efficacy was superior to that of selumetinib, an MEK inhibitor approved for treating patients with ESCC.

Using mass spectrometry (MS)-based drug affinity responsive target stability (DARTS), a most recent study discovered that 20(S)-protopanaxadiol showed strong binding affinity for PURα protein (Chen et al. 2023). According to molecular docking, 20(S)-protopanaxadiol bound to S102 and M103 residues of PURα, thereafter increasing the mRNA levels of PURα target genes in rat brains. The findings implied that 20(S)-protopanaxadiol might be a promising PURα agonist and the potential anti-tumor function of 20(S)-protopanaxadiol was supposed to be evaluated on cancers where PURα was discovered as a tumor suppressor in preclinical studies.

## Translational relevance

Dysregulation of PURα has been observed in many cancer types, including PC, BC, and ESCC. In preclinical settings, knockdown or overexpression of PURα has shown a great impact on cancer cell proliferation, drug resistance, and metastasis. These studies suggest that PURα possesses huge potential as a clinical biomarker and therapeutic target.

*PURA* gene deletion is frequently observed in MDS and is involved in progression to AML (Lezon-Geyda et al. 2001). A high frequency of *PURA* gene deletion is also found in MN and is associated with anti-apoptosis (Adema et al. 2022). These studies imply that detecting *PURA* gene deletion may reflect cancer progression. However, no evidence exists that *PURA* gene deletion is associated with patient survival in these cancer types. Whether *PURA* gene deletion can be a biomarker for cancer needs further investigation. In PC and BC, studies have shown the downregulation of PURα protein expression in hormone therapy-resistant cells via IHC (Yu et al. 2023; Wang et al. 2008). On the contrary, IHC staining has discovered upregulation of PURα protein expression in ESCC, especially metastatic ESCC (Gao et al. 2021; Tian et al. 2022). Therefore, IHC detection of PURα protein is a putative clinical method to predict hormone therapy resistance and cancer cell metastasis in certain cancer types.

Targeting PURα remains a challenge now. For ESCC, G699-0288 inhibits the interaction between MEST and PURα, exhibiting a strong anti-tumor effect in the PDX model with low toxicity (Xu et al. 2023). Considering the overexpression of PURα in ESCC (Gao et al. 2021; Tian et al. 2022), G699-0288 is a promising drug for ESCC treatment and needs further clinical trials. On the other hand, as a PURα agonist, 20(S)-protopanaxadiol (Chen et al. 2023) is supposed to inhibit hormone therapy-resistant PC and BC cells which require further function studies in the preclinical models.

## Conclusions and perspective

The most recent studies have greatly extended our understanding of the dual roles of PURα in different cancer backgrounds. While the function and dysregulation of PURα in AML, BC, PC, and ESCC have been revealed, it remains unknown how PURα contributes to the initiation and development of other cancers. Techniques such as LACE, seCLIP, and PAR-CLIP (Su et al. 2021; Blue et al. 2022; Danan et al. 2016), which identify the direct interaction of RNA and protein with nucleotide-level resolution in vivo, will provide novel insights for unveiling the RNA targets of PURα in cancer cells. As for the transcription factor function of PURα, new approaches such as CUT&Tag and DAP-Seq (Bartlett et al. 2017; Kaya-Okur et al. 2020) might uncover the transcription activator and repressor functions in cancer cells.

DNA/RNA binding proteins are known as “undruggable” targets, and developing PURα inhibitors or agonists remains challenging. Until now, only a few compounds targeting PURα have been discovered using molecular docking or DARTS (Xu et al. 2023; Chen et al. 2023). High-throughput small molecule screens based on fluorescence polarization and Föter resonance energy transfer (Henley and Koehler 2021) will help discover novel drugs for modulating PURα in cancer cells. Proteolysis-targeting chimera (PROTAC) is another alternative way to downregulate target proteins via promoting interaction between target proteins and E3 ligases (Sakamoto et al. 2001). Recent studies have shown that PROTAC downregulates the “undruggable” targets with satisfied anti-tumor effects (Adams et al. 2023; Xiao et al. 2022). For PROTAC application to PURα, further research is needed to uncover E3 ligases for regulating PURα protein stability. Targeting PURα is supposed to provide therapeutic approaches to improve the efficacy of hormone therapy, chemotherapy, and other first-line treatments for patients with cancers by inhibiting the emergence of resistant and metastasis cells.

Altogether, the current findings demonstrate a dual role of PURα in cancers. In situ hybridization detection of *PURA* gene deletion and IHC detection of PURα protein expression are valuable diagnostic approaches for MN, PC, BC, and ESCC. In addition, preclinical and clinical investigations of discovered agents targeting PURα will provide novel treatment strategies for patients. Future studies will unveil the complex roles and molecular mechanisms of PURα and give rise to new drugs to combat cancers driven by the dysregulation of PURα.

## Data Availability

No datasets were generated or analysed during the current study.

## Abbreviations

MDS: Myelodysplastic syndromes
AML: Acute myeloid leukemia
MN: Myeloid neoplasia
PC: Prostate cancer
BC: Breast cancer
ESCC: Esophageal squamous cell carcinoma
GBM: Glioblastoma
PURα/β: Purine rich element binding protein A/B
MBP: Myelin Basic Protein
HRPC: Hormone-refractory prostate cancer
IHC: Immunohistochemistry
AJCC: American Joint Committee on Cancer
TSS: Transcription start site
OS: Overall survival
HNRNPK: Heterogeneous Nuclear Ribonucleoprotein K
MEST: Mixed epithelial and stromal tumor
AGPG: Actin Gamma 1 Pseudogene
SG: Stress granule
MAPK: Mitogen-activated protein kinases
GC: Gastric cancer
AR: Androgen receptor
IP: Immunoprecipitation
MS: Mass spectrometry
CDK: Cyclin/cyclin-dependent kinase
IGFBP3: Insulin-Like Growth Factor Binding Protein 3
3’UTR: 3’ Untranslated region
CASP8: Caspase 8
PCK2: Phosphoenolpyruvate carboxykinase 2
SRCIN1: SRC Kinase Signaling Inhibitor 1
CLIP-seq: Cross-Linking and Immunoprecipitation sequencing
RASAL1: RAS Protein Activator Like 1
ELISA: Enzyme-linked immunosorbent assay
LACE: Linear amplification of complementary DNA ends
seCLIP: Single-end enhanced CLIP
PAR-CLIP: Photoactivatable-ribonucleoside-enhanced CLIP
CUT&Tag: Cleavage under targets and tagmentation
DAP-Seq: DNA affinity purification sequencing
PROTAC: Proteolysis-targeting chimera
